# A review on the relation between simulation and improvement in hospitals

**DOI:** 10.1186/1472-6947-12-18

**Published:** 2012-03-14

**Authors:** Wineke AM van Lent, Peter VanBerkel, Wim H van Harten

**Affiliations:** 1 Netherlands Cancer Institute - Antoni van Leeuwenhoek Hospital, Division of Psychosocial Research and Epidemiology, Amsterdam, The Netherlands; 2 Department of Health Technology Services Research (HTSR), University of Twente, Enschede, The Netherlands; 3 Department of Industrial Engineering, Dalhousie University, Halifax, Canada

**Keywords:** Simulation, Operations management, Implementation, Process improvement, Hospitals

## Abstract

**Background:**

Simulation applications on operations management in hospitals are frequently published and claim to support decision-making on operations management subjects. However, the reported implementation rates of recommendations are low and the actual impact of the changes recommended by the modeler has hardly been examined. This paper examines: 1) the execution rate of simulation study recommendations, 2) the research methods used to evaluate implementation of recommendations, 3) factors contributing to implementation, and 4) the differences regarding implementation between literature and practice.

**Results:**

Altogether 16 hospitals executed the recommendations (at least partially). Implementation results were hardly reported upon; 1 study described a before-and-after design, 2 a partial before and after design. Factors that help implementation were grouped according to 1) technical quality, of which data availability, validation/verification with historic data/expert opinion, and the development of the conceptual model were mentioned most frequently 2) process quality, with client involvement and 3) outcome quality with, presentation of results. The survey response rate of traceable authors was 61%, 18 authors implemented the results at least partially. Among these responses, evaluation methods were relatively better with 3 time series designs and 2 before-and-after designs.

**Conclusions:**

Although underreported in literature, implementation of recommendations seems limited; this review provides recommendations on project design, implementation conditions and evaluation methods to increase implementation.

**Methods:**

A literature review in PubMed and Business Source Elite on stochastic simulation applications on operations management in individual hospitals published between 1997 and 2008. From those reporting implementation, cross references were added. In total, 89 papers were included. A scoring list was used for data extraction. Two reviewers evaluated each paper separately; in case of discrepancies, they jointly determined the scores. The findings were validated with a survey to the original authors.

## Background

The median spending on healthcare in the Organisation for Economic Co-operation and Development (OECD) countries is 8.8% of GDP, with the USA spending 15% [1]. These countries are struggling to contain costs, forcing hospitals to rethink strategies on efficiency and the organization of processes. Due to the complexity and variability of many processes, managers find it difficult to estimate whether a redesign will result in significant improvements. To overcome this, hospitals need techniques that support them in making well-informed decisions about the trade-off between costs and quality [2]. Simulation provides various techniques that help hospitals face these challenges [3,4].

The perceived advantages of simulations and the growing number of applications [5] suggest this is a suitable approach for the healthcare sector. After reviewing 163 papers on operations research (OR) in healthcare Brailsford et al.[6] concluded that simulation is the second most popular OR technique after statistical analysis. These results are comparable with other sectors [7,8].

### The implementation of simulation recommendations

In the past 30 years, at least seven reviews on simulation in healthcare appeared [5,6,9-13], leading to the conclusion that simulation is widely used and can be regarded as a mature tool [14]. Four papers researched the prevalence of implementing recommendations derived from OR models in healthcare [6,9,13,15].

After reviewing more than 200 papers on simulation, Wilson [13] found only 16 studies reporting the execution of recommendations and only 11 of these papers described operational problems in healthcare. Lagergren found that almost two-thirds of the papers on OR models in healthcare discussed general OR aspects or did not report on execution [15]. In a review of 182 papers about simulation modeling in population health and health care delivery, Fone et al. [9] concluded that evidence of implementation is scarce. More recently Brailsford et al. [6] presented an extensive literature review of different types of modeling efforts in healthcare. They examined implementation on a three level scale: suggested (theoretically proposed by authors), conceptualized, implemented (actually used in practice). Only 5.3% of the 342 papers reported to have been used in practice.

Although these reviews provided insight into the prevalence of implementing simulation recommendations, it remains unclear if the results are valid in the present context. Wilson's study [13] although comprehensive, was completed in 1981, which leads to the question whether the conclusions are still valid, given the advances in simulation software and techniques. The review of Lagergren was not focused solely on simulation and was by the author's own account "incomplete" [15]. Fone et al. [9] reviewed simulations on population health and healthcare delivery instead of operations management in individual hospitals. Although the work of Brailsford et al. [6] appeared comprehensive, the results were not limited to simulation and did not examine the realized impact of the changes recommended by the simulation study.

We conclude that the reported implementation rates are low, and that none of the mentioned reviews examined the realized impact of the changes recommended by the modeler. This suggests that although simulation is widely reported upon in healthcare, it is not clear whether actual implementation is carried out by management.

### Realizing improvements with simulation models

To achieve improvements with simulation studies, one needs both a competent change management strategy and a simulation model and results deemed acceptable by the stakeholders. This paper focuses on simulation models. For references on change management, sufficient papers are available [16-19].

We identified two frameworks on the development of simulation models in healthcare [20,21]. Both emphasized that in healthcare the problem definition phase consumes more time due to conflicting stakeholder objectives and an unclear problem understanding. Additionally, more involvement of staff is required because decision makers are often unfamiliar with simulation techniques and therefore treat it with suspicion.

Understanding the relation between simulation models and improvements requires insight into the conditions that increase the implementation rate of recommendations. To our knowledge, four papers [22-25] have strived to identify these conditions. However, the focus and results were not specific to the healthcare sector. Robinson and Pidd interviewed 10 simulation modelers and 10 organizations to determine factors considered as important [24]. This led to SIMQUAL [25], a survey that compared the expected quality with the perceived quality. McHaney and Cronan used a contingency model of simulation success as input for a survey among 126 projects to examine the relation between simulation project characteristics and their success in 126 projects [23].

### Research objectives

Several research gaps exist on the relation between simulation applications in healthcare and the execution of the recommendations. Therefore, we report on the following research objectives:

1. To determine the frequency that simulation recommendations are executed to improve operations management in individual hospitals.

2. To determine what factors contribute to the implementation of simulation study recommendations.

3. To determine the research methods used to evaluate implemented simulation recommendations.

4. To examine the difference between literature and reality with regard to the implementation of simulation recommendations.

The answers to these questions can support the transformation from simulated scenarios to improved hospital processes.

## Results

**Figure 1:**
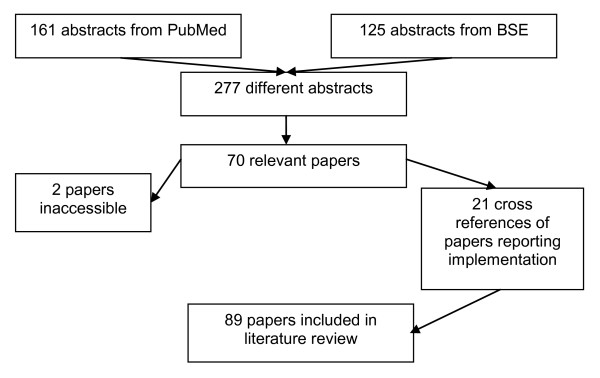
**Overview selected papers for literature review**.

The literature search strategy resulted in 161 abstracts in PubMed and 125 in Business Source Elite (BSE), in total 277 different abstracts. The reviewers selected 113 abstracts for inclusion. We obtained the full text of all, except 2 papers. Only 68 met all inclusion criteria. The cross reference check on those partially implemented simulation recommendations, resulted in 21 additional papers. In total, 89 papers were included. Figure 1 visualizes the paper selection process. The methods section in the end of this manuscript describes the details of the search strategy and the inclusion process.

### Section I: Project scope and background

Altogether, 68 papers simulated a single department, 21 multiple departments. Of all multi-department simulations, 12 included the nursing wards, 11 the operating theatre, 7 the emergency department, 7 diagnostic facilities, 3 the intensive care and 1 the pharmacy. The most frequently examined single departments are the emergency & accidents department (18 papers), the operations theatre (15 papers) and the consultations department (13 papers). Additionally, 9 papers reported the use of their model in more than one setting.

### Section II: Implementation phases

Table 1 shows that the simulation models of 73 papers presented (partial) direct benefits to the hospital and 26 stated that the hospital (partially) accepted their results. Only 10 papers reported the execution of recommendations, while 6 reported partial execution (totaling to 18%) and 3 mentioned the intention to do this.

**Table 1:** Results Section II: implementation phases

*Implementation phases*	*Yes*	*Partially*	*Intention is mentioned*	*No*	*Not stated*
Did the study achieve the clients objectives	89	0	N/A	0	0

Show the study results direct benefits to the client?	71	2	N/A	11	5

The study results are accepted by the client	21	5	N/A	0	63

The study results are executed	10	6	3	1	69

### Section III: Assessing the evidence that simulation leads to improvements

Only 3 papers reported data on the effects of the implementation. One of these papers had a before and after design (for definition see [26]) and 2 papers described a few results after the implementation. The post implementation measurements showed that the model of 1 paper was correct, whereas the other 2 papers were correct on most of the evaluated aspects.

### Section IV: Technical quality factors of a simulation study

Table 2 shows data availability is the most frequently mentioned factor that contributes to implementation of recommendations (57 times). The validation and verification of the simulation model through historic data was also identified as important (37 times). The same is true for validation and verification through expert opinion (30 times). Furthermore, the quality of the conceptual model was mentioned in 31 papers. The others' category mentioned the choice for modeling software and user friendliness 5 times, and modeler skills, 4 times.

**Table 2:** Results Section IV and V: Factors related to the technical quality and process quality of a simulation study

*Section IV: Technical quality factors *	*Times cited*
Data availability	57

Validation and verification through historic data	37

Quality of the conceptual model	31

Validation and verification through expert opinion	30

Keep the model as simple as possible	25

Quality of data	21

Quality of data analysis	21

Others	23

Model includes all relevant aspects	18

Sensitivity analysis	18

*Section V; process quality factors*	*Times cited*

Total commitment and support from user/client involvement	21

Appropriate use of animation in the model	19

Others	16

Communication between those involved	13

Well defined objectives and project scope	12

Complete the project within time	11

Realistic expectations between client and modeler	5

Do not exceed the available budget	2

### Section V: Factors related to the process quality of a simulation study

Table 2 also presents the process quality factors. Client involvement was the most frequently mentioned factor (21 times), followed by appropriate use of animation (19 times). In the others' category, 5 papers reported the importance of allowing sufficient time for the hospital to experiment with the model.

### Section VI: Factors related to the outcome quality of a simulation study related to implementation as stated by the authors

Most often (15 times), the presentation of the results was mentioned. Negative factors were: the simulated recommendations show improvements on one aspect but deterioration on others (10 times), changing circumstances during the project (5 times) and doubts on the cost benefit ratio (3 papers). In the others' category, 4 papers stated that, although relevant for implementation, a simulation model cannot include cultural or behavioral aspects.

### Survey

After a reminder we received responses for 41 papers; 29 returned the survey; for 12 papers the authors responded that they did not have the intention to improve a specific hospital process, the data only served to illustrate the potential of the model. The research thus limited itself to a method to calculate an optimal solution and in some cases involved a sensitivity analysis.

Table 3 shows that 21 of the 41 papers confirmed the (partial) acceptance of the recommendations. In 18 of the 41 papers (44%) the survey reported at least partial implementation of the results; the denominator (41), includes the categories missing, don't know, and not relevant. Additionally, the authors of 7 papers reported that the implemented changes were evaluated: 3 times with a time series designs (all from the same researcher), 1 with a controlled before and after design, 1 with a before and after design and 2 with partial before and after designs. Altogether the survey showed that in 9 papers the implementation proved the model to be correct, 5 other models were partially correct.

**Table 3:** Results of the electronic survey of the authors

*Question*	*Yes*	*Partially*	*No*	*Do not know*	*Missing *	*Not relevant for study*	*Total *
The study results are accepted by the hospital	11	10	1	4	3	12	41

The study results are executed	7	11	5	6	0	12	41

Implementation proved the study to be correct?	9	5	1	3	11	12	41

## Discussion

The literature review and the survey showed respectively an 18% and 44% implementation rate, suggesting that actual implementation occurred more often than reported in literature. Also the quality of the research methods in these cases was higher in reality (17%) than was reported in the literature (3%). Likewise the survey showed that more models proved to be correct in practice than in the literature. However, the survey reported that 14 models proved to be at least partially correct, while only 7 projects were evaluated with a before and after design. This casts some doubt on the reliability of the survey. It seems that some authors reported their model to be correct based on (subjective) reactions of the hospital.

An explanation for the differences between literature and practice is that the majority of papers focused on the technical simulation design and not the contribution to hospital improvements. This might be related to the authors' affiliations; the majority of the papers (66 out of 89) included at least one member of mathematical, operations research, industrial engineering or economics research groups. These researchers may prefer to publish technical modeling details. Consequently, most conditions for success were related to the technical quality of the model.

Fone et al. [9] provided another explanation; due to the time pressure to publish *"it is likely that many modeling studies are published before validation is complete and before implementation has been carried out (and assessed)."*

In addition, scientific publications on improvements achieved with simulation may be hampered because of the difficulty to draft a "ceteris paribus" design, to find control sites and of many unpredictable interfering variables.

Our study found that the majority of factors contributing to actual implementation concerned the technical quality of the simulation. Data availability was the most frequently mentioned factor (57 times). At least 43% of the involved hospitals had to generate new data to gain sufficient insight into their problem, this percentage could even be higher as 27% of the papers did not report on data collection. Data availability is important because the reliability of a simulation model is affected by the quality of the data used to calculate input distributions. Validation and verification of the models are essential to check the quality of the model (see Table 2).

Our study found that the process management factors are related to managing the expectations of the hospital and the modeler (see Table 2). This finding is consistent with Robinson & Pidd [24]. The use of animation was mentioned 19 times as a means to simplify communication between modeler and hospital. Animation, however, should be used carefully as it can distract staff from the model details [27]. Many of the process management factors are in agreement with Forsberg et al. [28], as they also identified cooperation, careful planning of the project and stakeholder and customer involvement as success factors for implementation of simulation models in healthcare.

Our finding that only 9 of the 89 papers reported the use of their model in more than one setting is in line with Proudlove et al. [29]. An explanation is that the emphasis placed on working closely with the client, meaning the best model for one hospital may be inappropriate for other hospitals [30]. Often researchers make detailed models to increase the statistical descriptive power, but this can hinder the demonstration of general principles [29]. Others [31] plead for more generic models after identifying differences between specific and general models in healthcare. In emergency care, initiatives have been undertaken to develop generic models [32].

### Research limitations

There may be a selection bias in the paper selection process as papers may be found in medical, health services and operations management and-research domains. We feel, however, that the included 89 papers are a good reflection of the available literature in this field. As this study focused on the implementation of recommendations and found a limited number of papers, we did not exclude papers because of the journals' impact factor or the quality of the models.

Although non-scientific literature contains many examples of simulation models in healthcare [6], we did not include these since non-peer reviewed articles are not held to the same rigorous quality standards. Additionally, it is difficult to systematically identify these publications [6]. Another limitation is the possible bias in respondents of the survey. We were only able to contact authors from 67 of the 89 papers. Furthermore, of the contacted authors related to the 67 papers, only 41 responded. It is more likely that staffs still present were involved in implementation.

### Future research

The relative advantage that an innovation (here simulation) has over other methods affects the uptake [33]. This paper found that implementation took place in 44% of the studies, however actual evidence that simulation leads to improved hospital performance is limited. To increase the uptake of simulation, researchers should provide high quality evidence of improvements. To get these results published, (scientific) journals could ask their authors to state whether or not the findings were accepted and implemented, and whether there is evidence of an improvement. A step further would be to have journals encouraging authors to submit follow-up papers describing the implementation of the recommendations. Furthermore, examining popular literature (e.g. trade journals, news media, etc.) on this subject remains an item for further research [6].

More research into perceived success factors seems necessary. Because of the encountered differences between literature and practice, it would be interesting to examine whether the technical, process and outcome quality of implemented recommendations are higher than those of studies that were not implemented. Furthermore surveys among multiple hospital respondents, such as management and medical professionals, that examine organizational characteristics contributing to the implementation of model findings seems worthy of further research.

This research was limited to simulation studies on operations management in hospitals. It would be interesting to extent the scope to other techniques to enable researchers to select the most appropriate OR techniques for specific settings. The overview of OR techniques and their advantages and disadvantages recently published by the RIGHT project is an important contribution [4] because it also discusses when to apply a specific technique and the required resources. In addition generalization of the methods and results needs further attention. It is relevant to identify a pool of generic approaches and to design a decision schedule for its use, involving the contingent factors relevant for the decision to embark on a specific simulation approach.

## Conclusions

This study showed that implementing recommendations of simulation applications on operations management in hospitals does not occur frequently; literature reports an 18% implementation rate and a survey among these researchers indicates a 44% implementation rate. Formal post implementation evaluations were hardly reported upon in literature; 1 study described a before-and-after design, 2 described partial before-and-after designs.

The lack of reported implementation of simulation recommendations might be explained by 1) academic factors: the authors' affiliations to mathematical/technical research groups, the time pressure to publish which is hampered by the long duration of implementation research and the difficulty to draft a "ceteris paribus" design, and 2) individual and organizational barriers that affect the development of the model and its implementation.

To ensure a wider uptake of simulation models in hospitals more evidence of improvements, based on rigorous evaluation methods, seems necessary. Modelers and their clients - in this case relevant users within hospitals such as physicians, nurses and managers- should pay more attention to the perceived success factors that affect the technical, process and outcome quality of the simulation.

Perceived success factors regarding the technical quality are data availability, validation and verification with historic data, validation and verification through (internal) expert opinion, and the development of the conceptual model to be used in the simulation. Client involvement is most important for quality of the development process. Presenting the results in an understandable and attractive way has a large impact on the usefulness of the model as it affects acceptance and actual implementation.

## Methods

A literature review on simulation applications in individual hospitals provided answers to all research objectives. Relevant literature was checked on: 1) the reported implementation of recommendations, 2) the evidence that the changes resulted in improvements and, 3) comments of the researchers on factors contributing to the implementation of the study recommendations.

In addition to the literature review, an email survey was sent to the traceable contact persons of the included papers.

### Search strategy and inclusion criteria

As we expect literature on simulation applications in individual hospitals to be found in healthcare and business literature we selected the Business Source Elite (BSE) and the PubMed database. Business Source Elite (BSE) covers 930 journals on business, management, economics, finance and related topics while PubMed contains biomedical literature from MEDLINE (biomedical literature) and life science journals. We used medical subject headings for the search strategy in PubMed, while for BSE we searched the abstracts for specific key words.

Two reviewers read all the abstracts and separately used the criteria of Table 4 to select papers for inclusion. We only included stochastic simulation applications that discussed operations management in individual hospitals and were published between 1997 and 2008. In case of disagreement, the full paper was retrieved and together a decision was made. Additionally, papers reporting (partial) implementation of the simulated recommendations were investigated further, with all cross references included according to the criteria listed in Table 4.

**Table 4:** Inclusion and exclusion criteria for abstracts

*Inclusion criteria*	*Exclusion criteria*
• The paper discusses an application of simulation • The discussed simulation model is stochastic. The current state does not determine the next state. • Goal of the simulation is to improve patient flow/process design or efficiency and resource capacity planning of primary processes (= processes related to patient care) • The simulation is concerned with processes within hospitals	• Other models than simulation • Deterministic simulation models • Applications outside the hospital • Simulations concerning processes in hospital systems in which multiple hospitals collaborate • Simulation models that support medical decision-making (related to guidelines), or preventing errors related to the treatment • No surveys and reviews • No staff rostering • Papers published before 1997 Papers written in other languages than English

### Data extraction

Using literature, a scoring list with multiple-choice answers was developed to analyze all papers. In a pilot, consisting of 10 papers, the form was adapted to its the final form.

The scoring list consisted of six sections. Section I asked for the simulated departments, the affiliation of the authors and the number of settings in which the model was used.

**Figure 2:**
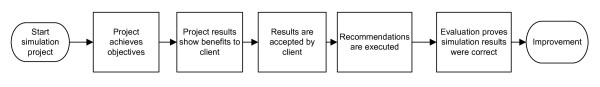
**Phases from simulation to improvement**.

Section II evaluated the extent of the implementation of the simulation. Figure 2 describes the implementation phases. The evaluation of the extent of the implementation was based on a four stage model of success in simulation studies [24]. We separated the first stage (the study achieves its objectives and/or shows a benefit) into two separate phases: project achieves objectives and project results show benefits to client. We consider this a necessary distinction since we expect projects that achieve their objectives, but do not show benefits to the client, will not be implemented.

Section III assessed the evidence that simulation leads to improvements (in case of implemented results) with the work of Eccles et al. [26]. It also examined whether implementation proved the results of the simulation to be correct.

Section IV, V and VI, concerned quality factors of the simulation study as stated by the author. We considered three quality aspects: technical quality, the process in which the model is developed, and outcome quality [34]. The latter concerns the usefulness of the simulation; did it support the decision making process? The factors related to each quality aspect were based on Robinson & Pidd [24].

Two reviewers evaluated each paper separately and in case of discrepancies, they jointly determined the scores; in case of disagreement the third author was involved.

### Survey

We were able to contact 67 of the 89 authors by email, the missing authors could not be traced. The survey consisted of 4 multiple choice questions that are comparable to the data extraction form. The survey asked the authors 1) whether the hospital accepted the recommendations of the simulation study 2) whether the recommendations were implemented, 3) whether the impact of the implementation was evaluated 4) whether the simulation study proved the recommendations to be correct.

## Competing interests

The authors declare that they have no competing interests.

## Authors' contributions

WvL and PV together developed together the design of the study and the analysis of the papers. WvL drafted the manuscript, PV contributed to revision of the manuscript. WvH was involved in the research design of the study, supervised it and contributed to the intellectual content of the manuscript. All authors read and approved the final manuscript.

## Pre-publication history

The pre-publication history for this paper can be accessed here:

http://www.biomedcentral.com/1472-6947/12/18/prepub
